# Myasthenia gravis, respiratory function, and respiratory tract disease

**DOI:** 10.1007/s00415-023-11733-y

**Published:** 2023-04-26

**Authors:** Nils Erik Gilhus

**Affiliations:** 1 Department of Neurology, Haukeland University Hospital, 5021 Bergen, Norway; 2 Department of Clinical Medicine, University of Bergen, Bergen, Norway

**Keywords:** Myasthenia gravis, Respiratory disease, Myasthenic crisis, Neonatal myasthenia, Ventilatory support, Comorbidity, Immunosuppressive treatment

## Abstract

Myasthenia gravis (MG) is characterized by muscle weakness caused by autoantibodies that bind to the postsynaptic membrane at the neuromuscular junction and impair acetylcholine receptor function. Weakness of respiratory muscles represents the most severe MG manifestation, and 10–15% of all patients experience an MG crisis with the need of mechanical ventilatory support at least once in their life. MG patients with respiratory muscle weakness need active immunosuppressive drug treatment long term, and they need regular specialist follow-up. Comorbidities affecting respiratory function need attention and optimal treatment. Respiratory tract infections can lead to MG exacerbations and precipitate an MG crisis. Intravenous immunoglobulin and plasma exchange are the core treatments for severe MG exacerbations. High-dose corticosteroids, complement inhibitors, and FcRn blockers represent fast-acting treatments that are effective in most MG patients. Neonatal myasthenia is a transient condition with muscle weakness in the newborn caused by mother’s muscle antibodies. In rare cases, treatment of respiratory muscle weakness in the baby is required.

## Introduction

Myasthenia gravis (MG) is an autoimmune disease where antibodies against the postsynaptic membrane at the neuromuscular junction lead to muscle weakness [35]. This muscle weakness is generalized in 80% of the patients, being localized to the ocular muscles in only 20%. Respiratory muscles can be involved, and also muscles important for laryngeal and pharyngeal function [43, 111]. Typical for MG muscle weakness is fluctuation over time and during the day. Repeated muscle use increases the weakness.

The MG autoantibodies are directed against the acetylcholine receptor (AChR) in 80% of the patients. Antibody binding activates complement and crosslinks the receptors, both mechanisms leading to AChR destruction and morphological changes with reduced folding in the postsynaptic membrane [40]. 1–10% of the patients have instead antibodies against muscle-specific kinase (MuSK), and a few have antibodies against lipoprotein-related peptide (LRP4). MuSK and LRP4 are associated with AChR in the neuromuscular junction, and these antibodies induce a reduced AChR function in the muscle membrane. At least half of the 15% of patients without muscle antibodies by routine testing have AChR, MuSK, or LRP4 antibodies with more sensitive tests [114].

MG has a prevalence around 250 per million in most countries and an annual incidence of 20 per million [4]. MG subgroups are defined from autoantibody specificity and generalized versus symptoms [43]. Age of onset and thymus pathology are additional criteria for the MG subgroups. A thymoma induces AChR antibodies and muscle weakness in 10% of the MG patients. Early-onset MG with debut before age 50 years and AChR antibodies typically have thymic hyperplasia, and surgical removal of thymus improves the muscle weakness. Juvenile MG with debut before age 16 years is rare in Europe and North America [86]. In China and other Far East countries, there is a subgroup of patients with debut before age 5 years [40]. This MG is usually mild, often ocular, respond well to treatment, and very rarely has any respiratory implications.

MG therapy includes symptomatic treatment with acetylcholine esterase inhibitor, thymectomy, immunosuppressive drug treatment, physical training, supportive therapy, treatment of comorbidities, and ventilatory support for severe cases. The majority respond well to treatment and have mild disease or are in pharmacological remission. However, 15–20% of MG patients are difficult to treat and continue to have moderate or even severe muscle weakness [74].

Respiratory function is critical for MG patients. Weakness in the diaphragm and other respiratory muscles represents the most severe MG manifestation. Such weakness can be chronic, but more dramatically appears as an acute exacerbation with rapid need for intensive hospital care. MG patients have a slightly increased mortality rate, in the period 1985–2005 it was 1.4 in Denmark [49]. Respiratory disease is a main contributor. Optimal respiratory function is crucial for work, leisure activities, and quality of life. In this article, we review clinically relevant aspects of respiratory function and disease in patients with MG. This includes direct consequences of the disease itself, of MG treatments, and of comorbidities.

## Methods

The purpose of this review was to implement specific information from recent, referee-based scientific journals on the topic respiratory disease and complications into the general evaluation of and treatment recommendations for MG. The databases PubMed and Web of Science were searched for the combination of “myasthenia gravis”, “respiratory disease” and “English” on October 20^th^ 2022. 1502 hits appeared in Pubmed. All 470 hits since 2016 were examined for title, 90 of these for abstract and 40 of these for full text. In Web of Science, the search was restricted to articles and gave 280 hits. Ten additional full-text articles from this database were examined. Single-case reports and experimental studies were not considered for this search. Further searches in the same databases were made for specific aspects addressed in this article. Secondary articles were found from the reference lists of the already examined articles. The selection of articles both for examination and for inclusion in the reference list was based on relevance for the aims of this review as well as on scientific quality. The author had already good knowledge of the general MG literature.

### Muscle weakness

Diaphragm, intercostal muscles, and accessory muscles contribute to respiratory movements. Laryngeal and pharyngeal muscles have indirect relevance for ventilation as their weakness can lead to aspiration, pneumonia, and impaired respiratory capacity. Most MG patients do not have clinical signs of respiratory muscle involvement [41]. However, in a Dutch-Belgian cohort of patients with generalized disease and AChR antibodies as many as 42% self-reported respiratory muscle weakness at baseline and 55% at maximum severity [25]. This probably represents an over-reporting as many patients with generalized MG have a feeling of fatigue with tiredness, lack of energy, and a wide spectrum of complaints ([94]. Risk factors for a severe form of generalized MG with AChR antibodies and respiratory involvement are thymoma, higher age, and presence of additional muscle antibodies such as against titin and ryanodine receptor [91, 92, 115]. Shortness of breath, i.e., a feeling of not being able to get enough air, can have many causes and does not necessarily mean hypoventilation or respiratory muscle involvement. However, new shortness of breath or a symptom worsening in a MG patient merits prompt examination.

MuSK antibodies are nearly always associated with generalized MG [27]. Swallowing difficulties are common in this MG subtype, together with dysarthria and facial weakness. Muscle atrophy occurs, in contrast to AChR MG [28]. MG patients with MuSK antibodies frequently have a weakness of respiratory muscles [90]. In a cohort from Italy, as many as 24 out of 75 patients (32%) had experienced at least one episode with the need for ventilatory support [28]. One year after onset, one-third of the Chinese patients with late-onset MuSK MG had respiratory involvement [113].

LRP4 MG tends to be mild, either with ocular weakness only or a mild generalized disease [115]. MG patients with antibodies exclusively against LRP4 very rarely have any respiratory muscle involvement.

### Respiratory muscle testing

The assessment of MG patients for respiratory function and muscle strength involves patient history, clinical examination, and supplementary tests. The symptoms of hypoventilation due to respiratory muscle weakness are unspecific, such as sleep disturbances and day-time sleepiness and headache. Shortness of breath can have many reasons in MG patients and is not a specific marker for muscle weakness. Diaphragmatic dysfunction is not a presenting symptom for MG [104].

The testing differs whether the patient experiences an acute and life-threatening exacerbation, or the situation is chronic and stable.

Deficits and symptoms in MG can be measured using validated clinical scales. These scales were developed for clinical studies but are increasingly being used in the routine clinical assessment of MG patients as well [102]. Scales with the focus on patient-reported data usually contain questions regarding respiratory function. MG-ADL (myasthenia gravis activities of daily living) includes a single question about breathing, with the alternatives normal, shortness of breath with exertion, shortness of breath at rest, and ventilator dependence (Table 1) [110]. It contains a similar question about ability to swallow. For measurement of muscle strength, QMG (quantitative myasthenia gravis) is most widely used. Forced vital capacity is scored as no, mild, moderate, or severe impairment according to fixed limits (Table 2) [10]. The MG Composite combines items from the two other scales and includes the same question as in MG-ADL, but specifies that the impairment should be thought to be caused by MG [15]. The MG Impairment Index includes one breathing question, similar to MG-ADL [9]. MG-QOL15r (quality of life, revised) does not directly reflect respiratory function [16]. These scales do not represent good instruments for the assessment of respiratory function and respiratory muscle strength in MG. They give better information on swallowing and risk for aspiration. In a recent review, the authors concluded that MG scales for the objective assessment of respiratory and bulbar functions are still lacking [102].

**Table 1:** Myasthenia gravis activities of daily living (MG-ADL) standardized score sheet [110]

Grade	0	1	2	3
Talking	Normal	Intermittent slurring	Constant slurring	Difficult to understand
Chewing	Normal	Fatigue with solid food	Fatigue with soft food	Gastric tube
Swallowing	Normal	Rare episode choking	Frequent choking. Diet change	Gastric tube
**Breathing**	Normal	Shortness of breath with exertion	Shortness of breath at rest	Ventilator dependence
Impairment of ability to brush teeth or comb hair	None	Extra effort, no rest periods	Rest periods needed	Cannot perform
Impairment of ability to rise from a chair	None	Mild, sometimes uses arms	Moderate, always uses arms	Severe, requires assistance
Double vision	None	Occurs, not daily	Daily, not constant	Constant
Eyelid droop	None	Occurs, not daily	Daily, not constant	Constant

**Table 2:** The quantitative myasthenia gravis (QMG) test [10]

Test item	None 0	Mild 1	Moderate 2	Severe 3
Double vision on lateral gaze, secs	61	11–60	1–10	Spontaneous
Ptosis, upward gaze	61	11–60	1–10	Spontaneous
Facial muscles	Normal lid closure	Complete, weak, some resistance	Complete, without resistance	Incomplete
Swallowing water	Normal	Minimal coughing	Severe coughing	Cannot swallow
Dysarthria counting aloud to 50	None	At 30–49	At 10–29	Before 10
Right arm outstretched, secs	240	90–239	10–89	0–9
Left arm outstretched, secs	240	90–239	10–89	0–9
**Forced vital capacity, %**	> 80	65–79	50–64	< 50
Right hand grip, kg				
Men	< 45	15–44	5–14	0–4
Women	> 30	10–29	5–9	0–4
Left hand grip, kg				
Men	> 35	15–34	5–14	0–4
Women	> 25	10–24	5–9	0–4
Head lifted, secs	120	30–119	1–29	0
Right leg outstretched, secs	100	31–99	1–30	0
Left leg outstretched, secs	100	31–99	1–30	0

A single breath counting test is used to clinically assess respiratory capacity. This test can be performed on the telephone as well. In a recent article, the authors found that the test administered on the phone by a trained nurse had a sensitivity of 80% and a specificity of 60% in diagnosing an MG exacerbation [66]. The test was performed by the patient sitting upright, inhaling deeply, and then counting out loudly for as long as possible in their normal voice at a rate of two counts per second. Patients with counts less than or equal to 25 were considered likely to have MG exacerbation. Respiratory function testing by spirometry gives information about restrictive and obstructive patterns and is important in the evaluation of MG comorbidities that can impair respiratory capacity. Expiratory muscle strength is typically low in MG patients with mild or moderate generalized disease [18]. Maximal inspiratory pressure and maximal expiratory pressure tend both to be reduced in MG. This reduction is modest in patients with generalized disease, but more pronounced in those with severe MG. Results in MG patients are often reported to be 50–90% of expected values, with a higher reduction for expiratory pressure [18]. A reduction from previous values indicates an MG exacerbation. Treatment needs should be defined from spirometry results combined with the clinical evaluation. Although spirometry reflects functional exercise capacity, it does not provide discriminative diagnostic information regarding MG weakness [48]. Weakness in facial muscles may interfere with spirometry. Unexpected airway obstruction has been reported in some well-regulated and clinically stable MG patients without any obvious respiratory muscle weakness [26, 62]. Vital capacity in MG correlates less well to the need of mechanical ventilation than in other neuromuscular disorders [93]. Respiratory rate, work of breathing, phonation, oxygenation, and time trends in these variables are more important than absolute numerical test values.

Imaging of the chest by CT or MR is essential in MG to evaluate the thymus, especially to detect a thymoma, which occurs in 10% of MG cases. Imaging gives at the same time some information about any pulmonary comorbidity, this being relevant for respiratory function. The diaphragm is the most important respiratory muscle. Assessment of diaphragm function is often necessary to decide whether respiratory dysfunction is due to MG weakness or to comorbidities. Diaphragm position is highly variable on static imaging. However, hemidiaphragm elevation has a high sensitivity for diaphragm weakness, 90%, although a low specificity, only 44% [48]. Fluoroscopy can be applied to visualize and evaluate diaphragm movement, and also dynamic MRI [68]. Asymmetry is easier to detect than bilateral weakness. Phrenic nerve injury represents a possible complication to thymectomy, especially during removal of thymomas [32], and can occur both after videoscopic and open thoracotomies. Injury will lead to unilateral diaphragm paresis and add to any MG-induced respiratory weakness.

Ultrasound imaging can provide bedside assessment of the diaphragm. It is quick to perform and can easily be repeated, for example during MG crisis [46, 68]. Ultrasound imaging gathers qualitative information, whereas quantitative information about diaphragm function is much less reliable [68]. Recordings of diaphragm thickening during voluntary contraction, two-dimensional speckle tracking imaging, and shear wave elastography have been used to estimate diaphragm strength. The cranio-caudal movement of the diaphragm dome during quiet and forceful breathing can be measured and reflects diaphragm motion.

Electrophysiological studies can provide information about the phrenic nerve, the diaphragm, and neuromuscular transmission. The phrenic nerve can be stimulated near the clavicle [48]. There are several specialized recording techniques available. Such tests are not used as a routine neither for MG diagnosis nor follow-up during severe exacerbations. However, they may give additional diagnostic information in therapy-resistant cases with long-term need of respiratory support.

### Comorbidities

Comorbid conditions represent a major challenge for MG patients. They are especially important when they affect the respiratory system since MG has ventilatory impairment as a major risk. Respiratory function represents a key factor for MG daily functions, quality of life, and mortality risk. Several MG comorbidities are related to the MG, such as other autoimmune disorders and thymoma paraneoplasias. Some are due to the MG treatment, such as corticosteroid side effects. Both MG and immunosuppressive treatment may increase the risk for respiratory infections, and the consequences of such infections can be more severe in patients already impaired by their MG. Comorbidities unrelated to MG will still interact with the MG weakness.

A Danish registry study suggested three main reasons for an increased MG mortality rate of 1.4; respiratory impairment, treatment-induced disorders, and autoimmune comorbidity [49]. A similar Swedish study pointed to respiratory infections as a major cause of death, in addition to cancer in the MG subgroup with a thymoma [109]. Respiratory infections gave an increased death rate in Norwegian MG patients previously, but not so obvious in recent years [81].

MG patients have an increased risk for all other autoimmune disorders, and 7–15% have a second autoimmune disease [38, 57]. Disorders such as SLE and diabetes can indirectly influence respiratory function. Autoimmune interstitial lung disease occurs very rarely together with MG. Early-onset MG with AChR antibodies and thymic hyperplasia is the subgroup with most frequent autoimmune comorbidity. Thymoma MG has a specific pattern for autoimmune overlap, but this does not include the respiratory system.

Most thymomas in MG are asymptomatic apart from their paraneoplastic MG manifestations. However, large thymomas can mechanically impair respiratory function.

Chronic pulmonary disease was recorded in 11% of the patients at baseline in a recent British MG cohort, and this increased to 21% during the follow-up period [50]. Asthma was associated with risk of MG in a systematic review, and with a risk ratio of 1.38 [112]. Two cohort studies and three case–control studies were pooled in this meta-analysis.

Heart failure will lead to respiratory impairment. Cardiomyositis occurs more frequently in MG than in other autoimmune disorders [22, 77]. This is probably in part due to a cross-reaction against heart muscle of MG skeletal muscle antibodies against antigens outside the neuromuscular junction [101]. Dyspnea was the most common symptom in MG complicated with cardiomyositis [22]. Total cardio-pulmonary disease load can be high in elderly MG patients. Hypertension (38%), diabetes (20%), myocardial infarction (15%), and congestive heart failure (13%) occurred frequently in a British MG cohort [50].

Sleep disorders in neuromuscular disorders should be suspected to be caused by hypoventilation associated with the disease [23]. Obstructive sleep apnea in MG patients is associated with male sex and obesity in the same way as in non-MG [51], and the symptoms and signs are also similar.

### Respiratory tract infections

Three main characteristics for MG are muscle weakness, autoimmune pathogenesis, and long-term immunosuppressive treatment. All three aspects may predispose for infections in the respiratory tract, increase the severity and complication risk of such infections, and influence respiratory infection treatment and prophylaxis. Respiratory muscle weakness predisposes for infections in the lower respiratory tract, and weakness for swallowing implies a risk for aspiration [39, 67, 111]. A population-based study from Canada found that MG patients had a 39% increased risk for serious infections after adjusting for confounders [61]. Among nearly 4000 MG patients, one-third experienced a serious infection during a 13-year observation period (73 per 1000 person-years). Respiratory infection, and bacterial pneumonia in particular, was most common.

Respiratory tract infections have been suggested as a causal factor for MG through molecular mimicry and cross-reactivity of T cells, B cells, or antibodies against virus antigens and muscle antigens [7]. Furthermore, infections induce a polyclonal activation of immunoactive cells that can include autoreactive T and B lymphocytes [39]. When MG patients are asked for triggering events, they will frequently list respiratory tract infections [12]. MG debut has been described during or immediately after several types of viral infections, and with new appearance of neuromuscular junction antibodies [39]. Molecular mimicry, cryptic viral antigens, epitope spreading, bystander activation, and polyclonal activation have all been suggested as mechanisms for this MG induction [69]. It has been suggested, but never proven, that a respiratory tract virus might enter the thymus and thereby induce MG, Epstein–Barr virus being a candidate [19].

Respiratory tract infection represents a common cause of MG deterioration. Both patient-reported and hospital data find that around one-third of the MG relapses and of severe exacerbations with respiratory impairment are precipitated by a respiratory tract infection [12, 45, 75, 88]. No specific microorganisms have been implicated. Infection in the respiratory tract can also occur secondary to an MG exacerbation due to muscle weakness and secret stagnation.

The risk for all types of infections increases when on immunosuppressive therapy. MG patients receiving such treatment have an estimated risk increase of 20–50% for respiratory infections [39]. The risk is drug dose dependent. None of the immunosuppressive MG treatments acts specifically on the neuromuscular junction immune response. Drugs with a selective action in the immune system such as B cell/plasma cell inhibitors, complement inhibitors, and FcRn blockers still have consequences for the immune response against respiratory tract microbes.

Thymectomy as treatment of MG influences T cell immunity. A delayed response to viral antigens is seen in some patients, but none of the responses was abrogated [87]. MG patients with a thymoma have an increased risk for Mycoplasma pneumoniae infection [59]. The consequences for respiratory tract infection risk are unknown, and they are difficult to examine as all patients have MG with muscle weakness, most use immunosuppressive drugs, and they tend to be more cautious regarding preventive measures. In a compulsory and nation-wide Norwegian registry, we found that MG patients had an increased odds ratio of 1.25 for the use of anti-infectious drugs [5]. This could be due to an increased frequency or severity of infections, but also reflect use of antibiotics for infections that would not have been treated without any MG. MG patients with a thymoma have more frequent and severe respiratory tract infections [53].

Covid-19 disease represents a pandemic caused by the acute respiratory syndrome coronavirus 2 (SARS-CoV-2). Therapeutic management has been challenging due to lack of specific antiviral treatment. In some patient groups, there has been a high mortality rate, mainly due to antibody-mediated inflammation in the respiratory tract and with a cytokine storm. New mutations of the virus have appeared, and the infection rate continues to be high. The risk for covid-19 infection does not seem to differ in people with and without MG [17, 98]. This is true even for MG patients on immunosuppressive treatment [85]. A reason may be that MG patients take more precautions to avoid being infected [24]. However, among those infected with covid-19, MG patients tended to have a more severe infection. In a German MG cohort, 34% of the patients with a proven covid-19 infection were hospitalized, and 12% died. Immunosuppressive drug treatment was a risk factor for a severe disease course. In a registry-based US study, MG patients were more likely to be hospitalized than patients with other autoimmune disorders [63]. 39% were hospitalized when infected, 13% were transferred to an intensive care unit, 4% had a ventilator, and 11% died. Age over 75 years and dysphagia increased the mortality. In a Czech MG cohort with covid-19 infection, 38% had severe pneumonia and 11% died [60]. Long-term corticosteroid treatment in high doses, recent rituximab treatment, cancer, and older age were risk factors.

Covid-19 infection does not seem to induce a more severe MG [17]. No change in MG disease severity was seen before and during the pandemic [24]. Similarly, only 15% of Czech MG patients had an exacerbation of their MG during the infection [60].

Vaccination against covid-19 in MG patients is safe and does not lead to exacerbations [14]. In a UK population of 32 million vaccinations, it was a slightly higher risk of hospitalization or death from myasthenic disorders 15–21 days after the first dose of adenoviral vaccine, but no heightened risk with mRNA vaccines [14, 82]. Several single-center studies have reported that covid-19 vaccination is safe in MG and should be strongly recommended [29, 73, 84]. In a review of 29 studies, the authors concluded that exacerbations and new onset of MG after covid-19 vaccination in a small proportion could not be ruled out [83]. However, publication bias has probably influenced this conclusion as several case studies were included, and also recall bias in the retrospective studies. Another review concluded that new-onset post-vaccine MG was mostly anecdotal, that no clear association between MG exacerbations and most vaccines was found, and that vaccines should be recommended to MG patients without any doubt [95].

Vaccination against influenza virus is safe in MG, given a strong antibody response, and should be recommended [14, 100]. The risk for aggravating MG is higher for influenza infection than for any vaccination [97].

### Neonatal myasthenia

Neonatal myasthenia occurs in around 10% of the newborn children of mothers with MG [8, 36]. The babies have transient muscle weakness that rarely affects the respiratory muscles. Only one of 15 children with neonatal myasthenia needed intubation for hypoventilation in a recent cohort [64]. More common symptoms are dysphagia, a weak cry, and sometimes also general hypotonia. Poor sucking combined with dysphagia leads to feeding difficulties and a risk for aspiration, with secondary respiratory problems.

All MG women should give birth in hospitals with experience in intensive care and mechanical respiratory support for the newborn. As the onset can be delayed, all MG newborns should be observed for 4 days for any respiratory weakness [36, 64]. Neonatal myasthenia is caused by mother’s muscle antibodies transported across the placenta during fetal life. The child has no MG-related antibody production and the myasthenic condition is transient lasting for up to a couple of weeks [37]. Neonatal myasthenia in a previous child increases the risk. MG severity in the mother does not influence the risk for respiratory weakness in the child. Neonatal myasthenia can be caused by antibodies to both AChR, MuSK, and LRP4. It may occur most frequently with MuSK antibodies [58]. Most cases of neonatal myasthenia are so mild that no treatment is needed. However, a baby with respiratory problems should be treated with a low dose of acetylcholine esterase inhibitor. Intravenous immunoglobulin and plasma exchange are additional options, in addition to intensive care including ventilatory support if necessary [36]. Respiratory insufficiency in a newborn due to transfer of mother’s AChR antibodies across placenta can appear even without an established MG diagnosis in the mother [36].

Arthrogryposis and fetal AChR inactivation syndrome are very rare conditions that can appear in children of MG mothers [36]. These conditions can impair respiratory function, either transiently after birth or long term. Mother’s IgG antibodies bind to the fetal type of AChR [20]. MG in mother is a rare cause of arthrogryposis [52]. In a group of 15 newborn babies with arthrogryposis that required ventilatory support, one had muscle antibodies transferred from the mother [11]. In contrast to the remaining 14, this baby improved spontaneously after some days and survived.

### Treatment

Symptomatic treatment with inhibition of acetylcholine esterase at the neuromuscular junction increases the local acetylcholine content and thereby improves respiratory muscle strength. A dose-related side effect of such treatment is increased secretion in the respiratory tract. The mucus can impair ventilation and increase the infection risk, especially if the respiratory muscles are weak. This is the reason why temporary withdrawal of acetylcholine esterase inhibitors is recommended when a patient needs ventilatory support during a MG crisis.

Immunosuppressive drugs and thymectomy improve the muscle strength in MG. No such treatments have a specific effect on respiratory muscles. Respiratory muscle weakness and a risk for hypoventilation increase the need for immunosuppression. If respiratory problems persist, there is a need for higher doses or more effective immunosuppressive drugs. Complement inhibitors and FcRn blockers represent new and effective immunomodulators. They are effective and safe in most MG patients who do not respond satisfactorily to standard treatments [54, 55, 96]. At present, these drugs are too expensive for ordinary use, and refunding policies vary between countries. An aim is to define MG subgroups where such expensive treatments can be approved. Acute or chronic respiratory muscle weakness of functional significance caused by muscle antibodies and not responding to standard treatments represents the most severe form of MG and an indication for high-cost treatment.

MG with comorbid respiratory tract disease represents a particular therapeutic challenge. Immunosuppressive drugs, in combination or at high dose, will improve the MG but at the same time increase the risk for respiratory infections. Frequent dose and drug adjustments are necessary for this patient group [39]. Intravenous immunoglobulin (IVIG) represents an effective treatment for MG exacerbations with no increased infection risk [40].

The benefits of physical exercise for chronic disease are well known, and this includes neuromuscular disorders. However, in MG the muscle weakness increases with repeated muscle use, and because of this “overuse weakness” MG patients are sometimes recommended to abstain from exercise. Several controlled studies have demonstrated that physical training programs are safe in MG, and that muscle strength and daily function improve [34]. Four studies including a total of 60 MG patients with mild to moderate disease have examined the effect of systematic respiratory muscle training for 4–12 weeks [30, 31, 56, 89]. The programs consisted of interval-based muscle endurance training combined with breathing retraining. Three of the studies reported improved respiratory muscle strength, and the MG score improved in one of the studies. The patients that completed the program reported that the training was safe and feasible, improved their physical fitness, and gave better quality of life. However, there were some dropouts. In one of the studies, the patients continued their training during 1-year follow-up [31]. Improved aerobic capacity and increased forced vital capacity have been found in MG patients after general physical exercise programs [21, 108]. A comprehensive training and rehabilitation program for MG patients during the 3 months before thymectomy reduced respiratory complications and need for intensive care after the surgery [3].

Mechanical insufflation-exhalation therapy with a cough-assist machine is helpful for patients with muscle weakness. In a study of 24 patients with neuromuscular disease, such therapy was effective and well tolerated in 11 [1]. The experience in MG is limited, but a cough machine may be tried in patients with respiratory impairment and stagnant bronchial secretions.

Infections as a cause of respiratory impairment in MG need fast and effective treatment. Even mild infections should be taken seriously. Antibiotic treatment should be given early. Fluoroquinolones, including ciprofloxacin and levofloxacin, should be used only with caution as they in rare cases have been associated with severe MG worsening [39]. The same is true for aminoglycosides and macrolides. Telithromycin should not be used in MG (Table 3). Drug-induced MG deterioration is rare, but possible for all new treatments. A general awareness of both patient and doctor is necessary. Most antibiotics are well tolerated by MG patients.

**Table 3:** Antibiotics to avoid in MG if possible (from [39])

Antibiotic drug
Telithromycin
Fluoroquinolones
Macrolides
Aminoglycosides

### Myasthenia gravis crisis

MG crisis is defined as the need of ventilatory support due to weakness of respiratory muscles in a patient with MG. This is encountered in 10–20% of all patients with generalized MG at least once in their life [2, 44]. A crisis is more common during the first 2 years after diagnosis [103]. In some patients, the MG diagnosis has not been established before the patient needs ventilatory support because of muscle weakness [93, 99]. MG crisis occurs more frequently in elderly patients, and median age was 72 years in a recent German multicenter study [79]. Thymoma patients tend to have a more severe MG with a higher risk for crisis, whereas early onset MG with thymic hyperplasia only rarely needs ventilatory support. Some patients have recurrent crises. Respiratory infections can precipitate an MG crisis [78]. MG hospitalizations were most frequent during the summer in USA [47], possibly due to heat-induced impairment of neuromuscular transmission. Similarly, an unpublished study from Turkey found that MG crisis was most common during the hot summer months. MG with MuSK antibodies represents a severe form of MG. However, also for this group, there has been a reduction in MG crisis risk with an earlier diagnosis and more effective long-term immunosuppressive therapy [27].

In a large registry-based study from USA, the MG crisis mortality was 4.5% [2], whereas it was 12% in a study from Germany [79], and 18.6% in a Chinese study [72]. The differences can at least in part be explained by differences in inclusion criteria. MuSK antibodies are associated with a worse outcome in MG crisis [65]. Comorbidities represent a risk factor during MG crises, and disorders in the respiratory tract in particular [79].

MG crisis is a reversible condition where immediate symptomatic and immunoactive treatment is needed. Median duration of mechanical ventilation was 12 days [79]. Age, late-onset MG, severe MG before crisis, and number of comorbidities represented risk factors for prolonged ventilation and stay in intensive care unit [71, 79].

**Fig. 1:**
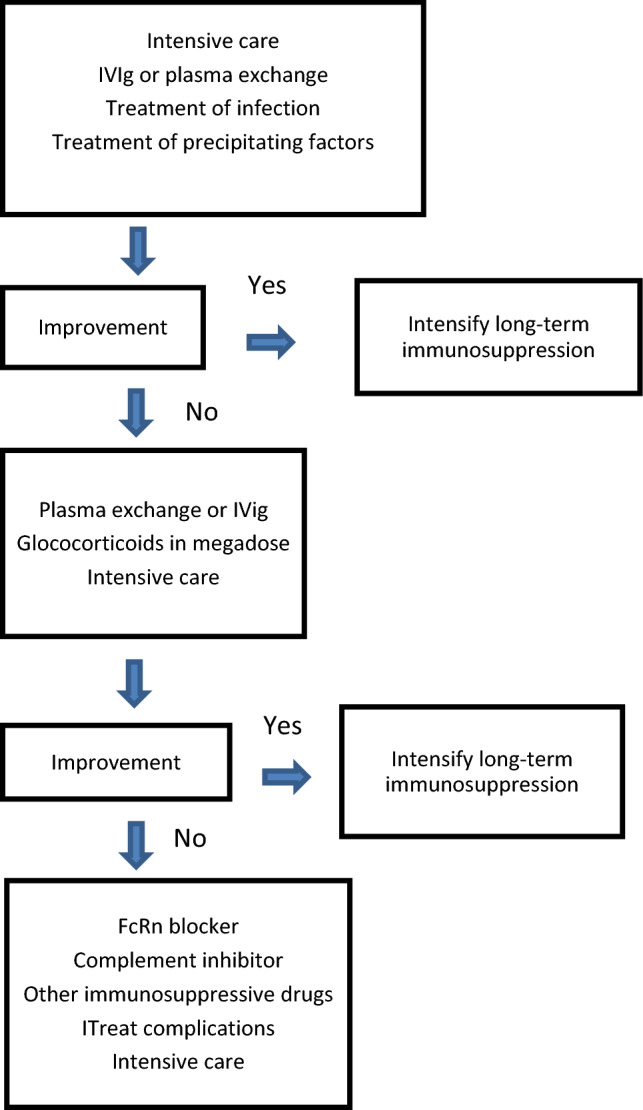
Treatment algorithm for acute exacerbations of myasthenia gravis with respiratory impairment. Adapted from [40]

The main symptom of an imminent MG crisis is progressive weakness of respiratory and bulbar muscles. Early and immediate hospitalization with admission to an intensive care unit is necessary (Fig. 1). Respiratory support can in some patients begin with non-invasive positive-pressure ventilation [76, 93, 106]. This was sufficient in one-third of a German cohort [79]. However, early intubation is needed for most patients due to the risk of aspiration and the rapid deterioration in respiratory muscle strength. One fourth of a Chinese MG crisis cohort and one-half of a German group received tracheostomy [6, 72]. Early tracheostomy was associated with shorter ventilation time and duration of intensive care unit stay.

Mechanical ventilation is the life-saving treatment in MG crisis. Any precipitating infections should be treated, as well as all other contributing comorbidities. Acetylcholine esterase inhibitors are temporarily stopped to reduce bronchial secretions [106]. IVIG and plasma exchange are regarded as equally effective to treat an MG crisis [33, 107]. Up to 80% of the patients respond to both treatments, but there is not a complete overlap between patients responding to the two treatments. The treatments can be repeated if only partial response. Megadoses of corticosteroids are sometimes added and with an effect after 2 weeks. Complements inhibitors and FcRn blockers are fast-acting drugs with an effect on therapy-resistant MG. They should be considered an alternative for difficult-to-treat MG crisis even if formal studies are still lacking [105]. In most patients, the long-term immunosuppressive treatment should be escalated after a MG crisis. Anxiety, depression, and fear of a new crisis is common in MG patients 1 year after an episode of respiratory insufficiency [70].

## Discussion

Respiratory muscle weakness represents a key determinant for MG severity and prognosis. The increased mortality rate in MG [49] is for a large part due to MG crisis and respiratory comorbidity. Similarly, acute hospitalizations for MG is most often caused by respiratory infection with exacerbation of general muscle strength and with a risk of hypoventilation [2]. Reduced respiratory capacity in MG influences daily-life functions, impairs quality of life, and increases the anxiety for severe exacerbations [42]. MG with MuSK antibodies, thymoma MG, older age, and respiratory tract comorbidities all represent risk factors for insufficient ventilation and a severe generalized MG [43].

There are several reasons why MG patients need improved treatment. Most patients have some remaining muscle weakness, many have a reduced quality of life, and they frequently experience side-effects of their current treatment, corticosteroids in particular [13, 42]. However, the most urgent need is for those with respiratory muscle weakness and threatening MG crisis. Complement inhibitors and FcRn blockers are at present too expensive to use for ordinary MG treatment but may be indicated for this MG subgroup.

MG crisis represents a threat for all MG patients. This threat is a reason for long-term specialist follow-up. Immediate in-hospital observation is necessary when suspicion of decreasing respiratory muscle function. Precise parameters and tests should be applied, and knowledge about risk factors is helpful in evaluation of individual patients. The threshold for transfer to intensive care unit and mechanical ventilatory support should be low. MG crisis is reversible, it does respond to best treatment, although a minority of the patients need highly active and combined therapy for several weeks [72, 79, 93].

MG females in reproductive age worry about pregnancy, and a large proportion abstain from pregnancy or have fewer children because of their MG [80]. A main factor is potential consequences of their MG and their MG treatment for the child. Precise knowledge and information about neonatal myasthenia, arthrogryposis, and fetal AChR inactivation syndrome is, therefore, important.

## Conclusions

Respiratory muscle weakness represents a threat for patients with generalized MG. The most severe manifestation is MG crisis with the need of temporary, mechanical ventilatory support. Increasing respiratory muscle weakness requires emergency hospitalization and follow-up in an intensive care unit. Comorbidities of the respiratory system reduce daily functions and quality of life. Respiratory tract infections commonly induce MG exacerbations. Effective and active immunosuppressive and symptomatic MG treatment long-term is necessary to reduce the risk for respiratory involvement and MG crisis. Comorbidities should be treated, and prevented if possible, not least respiratory tract infections. Females in reproductive age need special considerations regarding respiratory function for them and their newborn babies. New and more effective immunosuppressive drugs are of particular importance for MG patients with impaired respiratory function.
